# Compliance of Venous Thromboembolism (VTE) Assessment in Older Adult Inpatient Unit

**DOI:** 10.1192/bjo.2025.10427

**Published:** 2025-06-20

**Authors:** Rosy Purakayastha, Bassem Elsayed

**Affiliations:** SABP, Surrey, United Kingdom

## Abstract

**Aims:** Venous Thromboembolism (VTE) is a preventable condition that significantly contributes to morbidity and mortality in hospitalized patients. In psychiatric inpatient settings, VTE incidence ranges from 2–12%, with a rate of 1.5 per 1000 admissions within the first three months of hospitalization. The National Institute for Health and Care Excellence (NICE) guidelines recommend that all mental health inpatients, including those on psychiatric wards, should undergo a VTE risk assessment upon admission. The aim of this project was to assess compliance with these guidelines in the Older Adult Ward at East-Mid Surrey (The Meadows).

**Methods:** The initial phase involved auditing the records of all inpatients at The Meadows to assess the rate of completed VTE risk assessments. Feedback was then collected from junior doctors, who are primarily responsible for performing the assessments during patient clerking. The feedback indicated that the main barrier to completing the VTE assessments was difficulty accessing the risk assessment tool on the electronic medical records system, SystemOne. To address this, several interventions were introduced:

Posters with flowcharts on how to access the tool and an admission checklist were placed in the doctors’ office.

Emails were sent to all junior doctors to remind them of the importance of completing the VTE assessment.

An announcement was made at the Local Academic Programme to emphasize the need for compliance with the guidelines.

Collaboration with IT led to making the VTE tool more easily accessible on SystemOne, reducing the number of clicks required.

A re-audit of patient records was conducted after these interventions.

**Results:** Initial Audit: Out of 23 admitted patients, only 4 had completed their VTE risk assessments.

Feedback from junior doctors:

90% were aware of the NICE guidelines, and 70% could find them in the Induction Package.

100% found the assessment relevant to their practice, but 70% struggled to access the tool on SystemOne due to excessive clicks.

80% of doctors reported completing the assessment in under 5 minutes.

Post-Intervention Re-audit: Of the 19 admitted patients, 17 had completed their VTE assessments, indicating a marked improvement in compliance.

**Conclusion:** The initial audit highlighted a gap in compliance with VTE risk assessments due to accessibility issues with the electronic tool. The implemented interventions, including enhancing tool access, raising awareness, and providing reminders, resulted in a significant improvement in assessment completion. These steps should be continued to ensure better patient safety and adherence to NICE guidelines.

